# Effects of prior exercise on glycemic responses following carbohydrate inges on in individuals with type 2 diabetes

**Published:** 2015-07-17

**Authors:** Gisela Arsa, Laila Cândida de Jesus Lima², Daisy Motta-Santos, Lucieli Teresa Cambri, Carmen Silvia Grubert Campbell, John Eugene Lewis, Herbert Gustavo Simões

**Affiliations:** 1 Department of Physical Education, Federal University of Mato Grosso, Cuiabá, Mato Grosso, Brazil; 2 Department of Physical Education, Catholic University of Brasilia, Brasília, Federal District, Brazil; 3 Department of Physiology and Biophysics, Federal University of Minas Gerais, Belo Horizonte, Minas Gerais, Brazil; 4 Graduate Program on Physical Education, Catholic University of Brasilia, Taguatinga, Federal District, Brazil; 5 Department of Psychiatry and Behavioral Sciences, University of Miami, Miller School of Medicine, Miami, Florida, United States

**Keywords:** brief exercise, glycemia, insulinemia, carbohydrate

## Abstract

**Background:**

Exercise is effective in reducing glycemia, especially when it is performed in the postprandial period. However, no consensus exists in the literature about the effect of exercise on postprandial glucose control when it is performed before carbohydrate consumption.

**Aims:**

The main aim was to determine whether 20 min of exercise performed prior to carbohydrate consumption reduces postprandial glycemic and insulinemic responses. A secondary aim was to analyze the effectiveness of short-term (10 min) exercise bout with respect to postprandial glycemia reduction

**Methods:**

Nine individuals with type 2 diabetes (54.9 ± 1.7 years; 30.7 ± 1.8 kg/m^2^; glycemia level of 167.0 ±10.6 mg/dL) participated in the study and underwent the following procedures: (a) an incremental test to determine the lactate threshold; (b) an exercise session for 20 minutes at moderate intensity (90% of the lactate threshold); and c) a control session. The last two sessions were randomized, and the participants were monitored during 135 minutes of post-exercise recovery. A standard meal was consumed two hours before the experimental procedures started. A dextrose solution was administered at 45 minutes of post-exercise recovery while monitoring glucose and insulin concentrations. At 135 min of post-exercise recovery, eight of the participants performed an additional 10-min exercise bout following induced hyperglycemia.

**Results:**

Exercise reduced glycemia (−46.6 ± 7.9 mg/dL) and the insulin/glucose ratio (from 1.73 ± 0.59 to 0.93 ± 0.22 µU/mL/mmol/L) during the first 45 minutes of post-exercise recovery. Glycemia was significantly increased after carbohydrate consumption, reaching its peak value at 105 minutes of post-exercise recovery (261.8 ± 15.8 mg/dL) or control (281.3 ± 13.4 mg/dL). There was no effect of the previous exercise in attenuating glycemia or reducing the area under the curve for glucose and insulin after carbohydrate consumption. However, the effectiveness of exercise in reducing glycemia was shown again when it was performed at the end of the experimental session, even in case of only a 10-min exercise (reduction of −44.5 ± 4.9 mg/dL).

**Conclusions:**

Twenty minutes of moderate exercise does not alter the kinetics or the area under the curve in terms of glycemia and insulinemia after subsequent carbohydrate consumption. However, moderate exercise, even if performed for only 10-20 minutes, is effective in reducing postprandial glycemia in individuals with type 2 diabetes.

**Relevance for patients:**

Moderate-intensity exercise, even of short duration, may benefit individuals with type 2 diabetes on blood glucose control. A fast reduction in postprandial glycemia can be obtained with only ten minutes of exercise that, in turn, may ameliorate some of complications associated with the disease.

## 1. Introduction

An improper lifestyle, especially sedentarism and poor eating habits, contributes to the development of chronic degenerative diseases [1], among which type 2 diabetes mellitus (T2D) [2]. T2D is highly prevalent worldwide [3], with insulin resistance and chronic hyperglycemia as the main features. To mitigate the complications associated with T2D, fasting and postprandial glycemia control are essential [4].

Throughout the day, blood sugar levels of a healthy person fluctuate within a normal range and rise after food consumption, which results in increased insulinemia and therefore glycemia regulation. However, the post-meal elevation of glycemia is exacerbated in people with diabetes. This condition is occasionally identified by an oral glucose tolerance test (OGTT), in which the glucose uptake response and insulin release into the bloodstream [5] are determined following ingesting a solution with a high glycemic index.

Hyperglycemia should be avoided, particularly by individuals with T2D, since it leads to endothelial-, renal-, and neuronal damage, resulting in decreased release of vasoactive substances [6], reduced glomerular filtration rate [7], and impaired neuronal activity/sensitivity [8]. Accordingly, hyperglycemia is associated with the development of arterial hypertension [9], increased oxidative stress [10], renal insufficiency, and autonomic and peripheral neuropathy [11].

The body’s metabolic responses further depend on the type and quantity of food-derived macronutrients and their glycemic index [12, 13], as well as physical exercise. Regular physical exercise contributes to improved glycemic control [14], chronically decelerates the rate of glycemia increase, and improves insulin sensitivity [15].

The beneficial effects of chronic exercise are a result of the sum of acute effects. A single session of physical exercise causes an increase in glucose uptake by tissues during both exercise and post-exercise recovery. Exercise therefore acts as a non-drug therapy and is able to maximize the effect of hypoglycemic drugs [16]. Moreover, acute exercise promotes other metabolic benefits such as lipemia reduction [17] and increases in antioxidant enzymes [18]. The cardioprotective effects of exercise, through their reduction in blood pressure and peripheral vascular resistance, are associated with better acute metabolic function after exercise [19]. Even a single exercise session may result in increased insulin sensitivity, lipoprotein lipase activity, nitric oxide release, and translocation of glucose transporter 4, altogether accounting for a reduction in hyperglycemia [20].

However, the few studies that analyzed glycemic and insulinemic responses after physical exercise and a subsequent meal were performed at large temporal intervals between the end of exercise and the meals [21-26]. These studies also used variable combinations of macronutrients, which may affect the glycemic and insulinemic responses due to the differential glycemic indices of the meals and times of gastric emptying [27-29].

In a study by Larsen et al. [23, 24], glycemia and insulinemia reductions were observed after moderate (45 minutes at 50% of the maximum oxygen consumption (VO_2max_) and intense exercise (4 series of 3 minutes at 56.5% of VO_2max_ and 4 minutes at 98.3% of VO_2max_) performed by individuals with T2D. However, this effect was not sustained after consumption of a small meal containing carbohydrates, fats, and proteins during the recovery period, i.e., four hours after the exercise. In the study by Derave et al. [21], men with metabolic syndrome received a standardized meal and were subjected to physical exercise on a cyclergometer (45 minutes at 60% of the peak rate of oxygen consumption (VO_2peak_). Two hours later, the participants exhibited reduced glycemia in response to the prior exercise. Following intake of a meal, the glycemic and insulinemic responses were similar to that observed in a 24-hour period without exercise.

On the other hand, Oberlin et al. [30] verified in T2D subjects that a single exercise session (60 min at 60-75% of HR reserve) lead to a reduction in glycemia during the first 24 hours of recovery as well as a reduction in the area under the glycemia curve two hours after each meal, starting from the second meal. In a study with a similar purpose, Rynders et al. [31] subjected pre-diabetic individuals to a moderate exercise session (40.1 ± 9 minutes at 50% of VO_2peak_) and conducted an OGTT after 60 minutes for 3 hours. An increase of 51% in insulin sensitivity was observed with significant reductions in the area under the glycemia and insulinemia curves in the last two hours of OGTT.

Generally, studies that evaluate the effects of aerobic exercise combine low intensity and long duration bouts (e.g., 45-60 minutes) performed on a cyclergometer or a treadmill. Studies evaluating glycemic responses to shorter exercises are necessary as individuals with T2D, who are commonly sedentary and exhibit low aerobic fitness, usually do not engage in physical exercise program lasting longer than 30 minutes [32].

Currently it is unclear whether short acute aerobic exercise is sufficient to reduce glycemia and to attenuate the increases in glycemia and insulinemia after subsequent carbohydrate consumption. Such response may improve the metabolic control in diabetics, possibly reducing secondary complications associated with the disease.

In this study, we investigated the effects of a single moderate aerobic exercise session of 20 minutes on glycemia and insulinemia before and after subsequent ingestion of a carbohydrate solution in individuals with T2D. In addition, we studied the efficacy of just 10 min of moderate exercise performed after hyperglycemic induction, on glycemia reduction. Our hypothesis was that the physical exercise session would not only reduce post-exercise glycemia, but would also increase insulin sensitivity and attenuate the increase in postprandial glycemia and insulinemia in the post-exercise period.

## 2. Participants and methods

The protocols used in the present study were approved by the Ethics Committee on Human Research (SES/DF#087/2007, Brazil) in accordance with the Declaration of Helsinki (1964). Prior to participation, all subjects received a full explanation about the study protocol and purposes and signed a written informed consent form.

### 2.1. Participants

Nine individuals with T2D, all sedentary, participated in the study. All participants were taking oral hypoglycemic drugs in singular or combined form, including sulfonylureas, metformin, metformin+glibenclamide, glimepiride, and pioglitazone chloridrate. None of the participants were undergoing insulin treatment. Five of the participants were taking anti-hypertensive medication such as calcium channel antagonists and diuretics. All medication was washed-out 48 h prior to the initial screening visit, and the three subsequent experimental sessions were conducted under medical supervision. The individuals were also asked to avoid physical exercises and ingestion of alcoholic or caffeinated drinks for 24 h prior to each visit to the lab. The exclusion criteria were: history of cerebral stroke or acute myocardial infarction, severe secondary complications (such as blindness or wounded diabetic foot), physical or cardiovascular problems that could impair the performance of exercise, target organ damage, and current or previous tobacco use, post-menopausal women, and an age of < 40 and > 60 years old.

### 2.2. Screening visit

After a 12-hr fasting period, body weight (kg), height (cm), and abdominal circumference (cm) were measured. Venous blood samples (10 mL) were obtained for insulin analysis. A capillary blood sample was collected for the determination of blood fasting glucose concentration (Accu-check Advantage, Roche, Germany). Glycated hemoglobin (HbA1c) was estimated as proposed by Rohlfing et al. [33]. The insulin resistance index, HOMA-IR (< 2.71), was estimated as proposed by Matews et al. [34].

### 2.3. Procedures

To meet our primary objective, participants underwent three experimental sessions at the laboratory under the supervision of a cardiologist: (a) a maximal incremental test (IT) to determine the lactate threshold (LT) and to establish the exercise intensity of the constant workload exercise session; (b) a constant workload submaximal exercise session (SE) at 90% LT; and (c) a control session (CS). The two last visits were randomized and performed with a minimum intersession interval of 72 hours. All sessions began at 8 AM. At the beginning of each experimental session, blood glucose was measured (Accu-Chek Advantage) and, if the values were outside the 100 mg/ dL and 300 mg/dL range, the experimental session did not occur and the session was rescheduled for the ensuing week [35].

To meet our secondary objective, eight (six men and two woman) of the nine participants performed 10 minutes of exercise on a cyclergometer after 90min of carbohydrate ingestion (at 135 minutes of recovery either from control or exercise session), thus the participants were hyperglycemic.

#### 2.3.1. Incremental exercise test and aerobic fitness assessment

An incremental exercise test (IT) was performed using an electromagnetic cyclergometer (Excalibur Sport, Lode, Groningen, the Netherlands) with one minute warm-up at 60 rpm without load followed by the addition of 15 W every 3 minutes. The criteria used for stopping the IT were volitional exhaustion or any alteration detected that could impair exercise continuity. The individual electrocardiogram was constantly monitored by a physician. As markers of aerobic fitness, both the LT and VO_2peak_ were determined. The VO_2peak_ was obtained through breath-by-breath gas analysis system (MetaLyser 3B, Cortex, Biophysik, Leipzig, Germany) considering the highest value of oxygen consumption in mL/kg/min, while the LT was extrapolated from blood lactate concentrations obtained from earlobe blood samples (YSI 2700 Select Biochemistry Analyzer, YSI, Yellow Springs, OH). Extrapolations were based on the workload (Watts) corresponding to the inflection point in blood lactate levels (mmol/L) vs. workload curve, as previously described [36].

### 2.4. Standard breakfast and dextrose drink solution

Prior to the experimental sessions, the participants (Table 1) received a standard moderate glycemic index breakfast. This meal had a glycemic load of 39.2 and a glycemic index of 73.9 [37], totaling 322 Kcal (5.7% or 4.6 g of protein; 26.6% or 9.5 g of fat, and 65.8% or 53 g of carbohydrates).

**Table 1.** Demographics, medical details, and exercise parameters of the type 2 diabetes participants. Data are expressed as mean ± standard error (N = 9).

Gender (male/female)	7/2
T2D diagnosis (years)	5.5 ± 1.2
Age (years)	54.9 ± 1.7
Body weight (kg)	90.0 ± 5.9
Height (m)	1.71 ± 0.02
Body mass index (kg/m)	30.7 ± 1.8
Abdominal cincumference (cm)	103.7 ± 4.7
Fasting glycemia (mg/dL)	167.0 ± 10.6
HbA_1C_ (%)	6.9 ± 0.3
HOMA-IR index	2.8 ± 1.5
VO_2peak_ (mL/kg/min)	20.1 ± 1.7
PPO (W)	110.0 ± 8.9
Lactate threshold (W)	65.8 ± 5.8
Lactate threshold (%PPO)	60.1 ± 2.2

Abbreviations: T2D, type 2 diabetes; HbA_1c_, glycated hemoglobin; HOMAIR, insulin resistance index; PPO, peak power output; %PPO, percentage of peak power output at which the lactate threshold was attained.

Two hours after breakfast, participants underwent either a control or exercise session, and after 45 minutes of post-exercise recovery (R45) a dextrose solution was given to the participants. This solution contained 0.5 g dextrose/kg body weight, diluted in a proportion of 1 g of dextrose for each 5 mL of water plus 10 g of natural lemon juice for flavor.

### 2.5. Submaximal exercise at constant workload and control session

**Figure 1.**
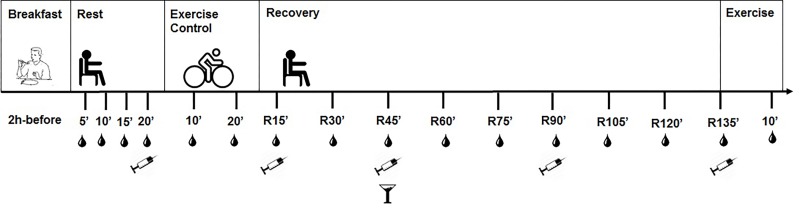
Summary of experimental design, entailing breakfast, a rest period, a 20-min submaximal exercises at constant workload (SE) or control session (CS) (both performed by all (9) participants), a recovery period, and a 10-min bout of moderate exercise (performed by 8 participants). Symbols: Drop: capillary blood collection for determination of glycemia. Syringe: venous blood collection for determination of insulin levels. Cup: administration of dextrose solution.

The experimental design of the exercise and control sessions is presented in Figure 1. The SE session was carried out on the cyclergometer and lasted for 20 minutes at 60 rpm. The intensity of cycling was set to 90% of the LT (90% LT). The CS followed the same pattern but with a seated resting period instead of the exercise bout. The SE and CS regimens had the following experimental design. In the pre-exercise period (rest), the participants remained seated and resting for 20 minutes. Heart rate (HR) (S810i, POLAR, Kempele, Finland) was measured every 5 minutes, and capillary and venous blood samples for blood glucose and insulin, respectively, were collected at the end of this period. Next, the exercise or control period was started and lasted 20 minutes. At the 10^th^ and 20^th^ minute of exercise (SE) or its corresponding resting time frame (CS), the HR, rating of perceived exertion [38], and blood glucose and lactate concentrations were measured. Finally, the recovery period was initiated immediately after the exercise period or control. The participant rested for 135 minutes (R15-R135) in a seated position in a quiet room. Capillary blood glucose was measured every 15 minutes and venous blood samples were obtained every 45 minutes during the post-exercise recovery period (R45, R90, and R135) for insulin analysis.

### 2.6. Single 10-min bout of moderate exercise after SE/CS recovery

After the 135-minute SE or CS recovery period, eight participants performed 10 minutes of exercise on a cyclergometer (Figure 1) to determine the effects on blood glucose levels. Capillary blood samples were obtained from the earlobe before and after 10 minutes of exercise for glycemia determination (mg/dL).

### 2.7. Blood collection and capillary, venous, and plasma analysis

In the SE and CS sessions, capillary blood samples were withdrawn from the earlobe after local antisepsis. The first blood drop was discarded to minimize sweat contamination, after which 25 µL of capillary blood was collected with a previously calibrated heparin glass capillary tube and transferred to Eppendorf tubes containing 50 µL of 1% sodium fluoride. The blood samples were stored at –20 °C until further use. Lactate (mmol/L) and glucose (mg/dL) were analyzed by an electroenzymatic assay.

The venous blood samples (10 mL) were obtained after local antisepsis from the antecubital vein. The blood sample was transferred to a dry glass tube and centrifuged at 3000 × g (LS3 Plus, Celm, Sao Paulo, Brazil) for 10 minutes. Plasma was aliquoted into Eppendorf microtubes and stored at –20 °C until further use. A chemiluminescence-based sandwich ELISA was used to analyze insulin (ACS 180 Automated Chemiluminescence System, Siemens Bayer, Munich, Germany) [39].

### 2.8. Data analysis

The total area under curve (AUC) was derived with the equation: Σ(*Area* 1 + *Area* 2 + *Area* 3 + *Area n*), whereby each area was calculated by: [(*larger base* + *smaller base*) × *amplitude* ]/2. For blood glucose, six areas were obtained using the amplitude at 15 minutes. For insulin levels, two areas were obtained using the amplitude at 45 minutes. The insulin/glucose ratio was expressed as (µU/mL)/(mmol/L).

### 2.9. Statistical analysis

**Figure 2.**
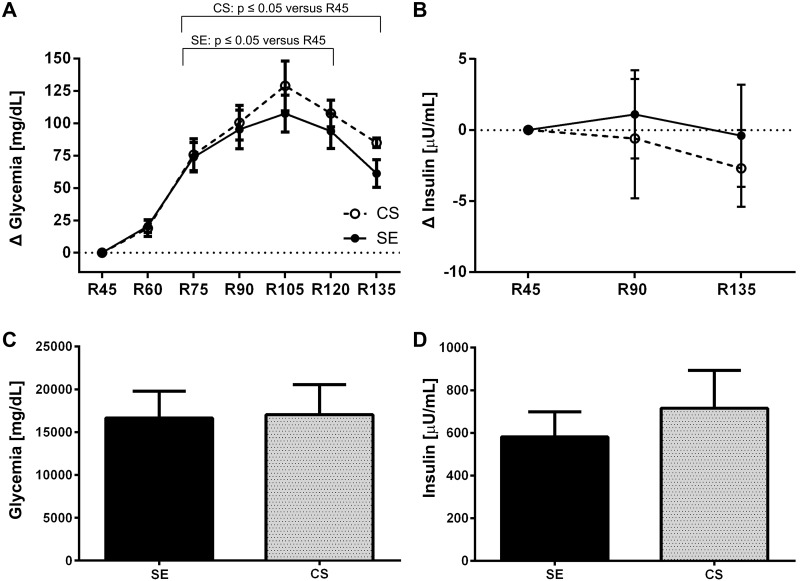
Glycemia (A) and insulin (B) kinects after dextrose ingestion by individuals with type 2 diabetes following 45 min of post-exercise recovery. The legend for panels A and B is provided in the bottom right corner of panel A. Total area under the curve for of glycemia (C) and insulin levels (D) for the R45-R135 period. Abbreviations: SE, submaximal exercise at constant workload; CS, control session. No differences were observed between the SE and CS at any of the time points.

**Figure 3.**
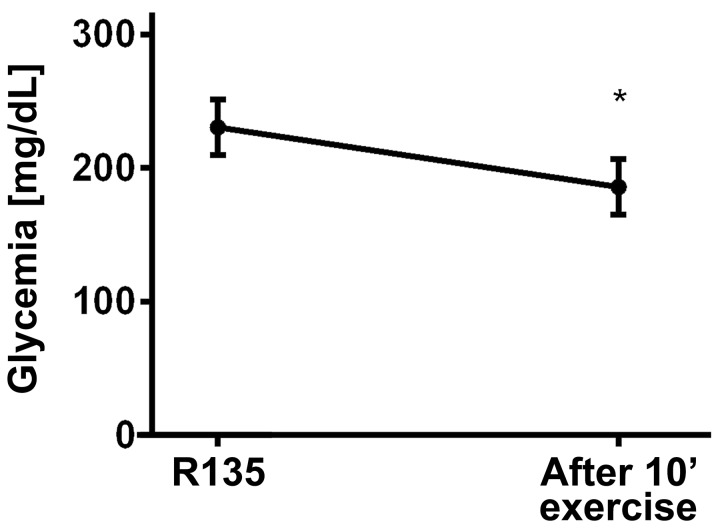
Glycemia (mg/dL) after 10 minutes of exercise after 135 minutes of post-exercise recovery and a dextrose challenge at 45 minutes in the recovery period. *p < 0.05 in relation to R135.

The data were expressed as means ± standard error of the means. The Shapiro-Wilk test was used to confirm a normal distribution of the data sets. A repeated measures ANOVA with Bonferroni post-hoc test was used to compare intragroup glycemia and insulinemia values at different time points (Figure 2A and B, Tables 3 and 4). An unpaired student’s t-test was used to compare the AUC for glycemia and insulinemia between the SE and CS sessions (Figure 2C and D). A paired student’s t-test was employed to analyze the effect of 10-min exercise, performed after hyperglycemia induction, on blood glucose control (Figure 3). The level of significance was set at p ≤ 0.05.

## 3. Results

### 3.1. Demographics, medical details, and exercise outcome parameters

The demographics, medical details, and exercise outcome variables of the study cohort are listed in Table 1. The T2D participants were obese and had an above-normal abdominal circumference, which are common features of T2D, and high fasting glycemia. The HOMA-IR index values were in the normal range, possibly due to the continuous use of T2D medication, which increases insulin sensitivity. These medications may exhibit sustained effects even after a 48-hour abstinence period. With respect to the IT parameters, the reduced VO_2peak_ reflects impaired aerobic capacity and reveals the group’s sedentary lifestyle.

### 3.2. Incremental exercise test and aerobic fitness status

Table 2 presents the variables related to the performance during exercise. The percent VO_2peak_, percent heart rate reserve, and rating of perceived exertion indicate that the submaximal exercise at constant workload session was performed at moderate intensity.

### 3.3. Glycemia and insulinemia kinetics before and after constant workload submaximal exercise and after dextrose administration during post-exercise recovery

The glycemia and insulinemia kinetics after the SE session at 65 ± 24 W or the CS and after consumption of a dextrose solution at 45 min post-SE/CS recovery are summarized in Table 3. Twenty minutes of moderate exercise reduced the glycemia to decrease in relation to pre-exercise resting period, which persisted up to 45 minutes of recovery when the participants ingested the dextrose solution. These data suggest that glucose was taken up from the circulation. Dextrose consumption led to an increase in glycemia, which peaked at R105 for both SE (261.8 ±15.8 mg/dL) and CS (281.3 ±13.4 mg/dL) (Figure 2A). The level of glycemia was significantly higher after dextrose consumption at R90-R120 compared to the glycemia level at resting state in the CS session (Table 3), demonstrating the effectiveness of exercise in controlling glycemia levels. No differences were observed between the SE and CS regimens (Figure 2A), also when the AUC was calculated (Figure 2C). With respect to insulin concentration, no differences were observed at any of the recovery time points compared to resting state or to the point before dextrose consumption (R45) in either experimental session (Table 3). Moreover, no differences were observed between the SE and CS regimens at any time point (Figure 2B and D).

**Table 2.** Performance variables during the 20-min constant workload submaximal exercise session. Data are presented as mean ± standard error.

Workload (W)	68.3 ± 6.7
VO_2peak_ (mL/kg/min)	13.7 ± 1.0
%VO_2peak_	9.2 ± 2.3
Heart rate (beats per minute)	114.7 ± 4.7
% Heart rate reserve	58.5 ± 0.4
Blood lactate (mmol/L)	3.0 ± 0.3
Rate of perceived exertion	13.6 ± 1.0

**Table 3.** Glycemia (mg/dL) and insulin (µU/mL) levels during the experimental sessions. Data are expressed as mean ± standard error.

	Glycemia		Insulin	
	SE	CS	SE	CS
Rest	201.0 ± 14.7	187.1 ± 11.8	17.0 ± 5.7	13.5 ± 4.7
Post	158.7 ± 16.1^a^	169.2 ± 15.3	7.0 ± 1.2	13.8 ± 4.2
Post at R45	154.5 ± 16.4^a^	158.9 ± 15.3		
^*^R45	154.3 ± 16.1ª	152.3 ± 14.9^a^	12.0 ± 2.8	15.9 ± 5.4
R60	174.7 ± 13.2	171.3 ± 15.8		
R75	228.2 ± 12.1^b^	228.1 ± 20.2^b^		
R90	249.6 ± 13.8^b^	252.9 ± 14.7^a^,^b^	13.2 ± 3.2	15.3 ± 3.0
R105	261.8 ± 15.8^b^	281.3 ± 13.4^a^,^b^		
R120	248.3 ± 23.0^b^	260.0 ± 20.1^a^,^b^		
R135	215.5 ± 24.5	237.2 ± 21.5^b^	11.4 ± 2.5	13.2 ± 4.3

Definitions: Post, measurement time point directly at the end of SE or CS;

Abbreviations: SE, submaximal exercise at constant workload; CS, control session

Notes: Post-R45: mean of values obtained at 45 min of post-exercise recovery; R45-R135: post-exercise recovery period.

*R45: measurement at the time of dextrose solution ingestion

^a^ p≤0.05 compared to Rest

^b^ p ≤0.05 compared to R45

**Table 4.** Insulin/glucose ratio for specific moments of the exercise and control sessions for individuals with type-2 diabetes.

	SE	CS
Rest	1.73 ± 0.59	1.28 ± 0.40
R15	0.93 ± 0.22^a^	1.56 ± 0.39
R45	1.53 ± 0.34	1.98 ± 0.58
R90	0.99 ± 0.22	1.05 ± 0.19
R135	1.35 ± 0.55	1.04 ± 0.28

Abbreviations: SE, submaximal exercise at constant workload; CS, control session

Note: R45 represents measurement after dextrose solution ingestion

^a^ p≤0.05 compared to Rest

### 3.4. Insulin/glucose ratio

Table 4 shows the insulin/glucose ratio at rest, after SE or CS, and following the consumption of dextrose solution at R45. For the SE group, the insulin/glucose ratio was reduced at R15 in relation to all other time points. After the dextrose consumption, the insulin/glucose ratio returned to baseline levels. No differences were observed between SE and CS at any of the time points (p>0.05).

### 3.5. Effect of a single 10-min bout of moderate exercise performed after hyperglycemia induction

Figure 3 shows the effect of a 10-min physical exercise bout on a cyclergometer (90% LT) performed by eight participants after 135 minutes of post-exercise recovery. This 10-min exercise bout reduced glycemia from 230.5 ± 78 mg/dL to 185.96 ± 77.3 mg/dL, corresponding to a decrease of 44.5 ± 4.9 mg/dL (19%), which suggests that short duration moderate exercise is effective in controlling postprandial glycemia.

## 4. Discussion

This study investigated the effects of a single moderate aerobic exercise session of 20 minutes on glycemic and insulinemic responses before and after a subsequent consumption of a carbohydrate solution in individuals with T2D. In addition, the efficacy of just 10 min of moderate exercise on blood glucose control was determined after hyperglycemia induction in the recovery period. Our results demonstrate that, although prior exercise was effective in reducing glycemia and the insulin/glucose ratio during the first 45 minutes of post-exercise recovery, it was not effective in reducing the glycemic and insulinemic responses subsequent to the dextrose consumption in these participants.

The reduction in glycemia after a single exercise session observed in our study is supported by other studies, where the exercise duration was similar to or higher than ours, as for instance employed by Poirier et al. [26] and Hiyane et al. [16], who exercised individuals with T2D. Both studies showed a hypoglycemic effect of physical exercise. The present study demonstrated that even just 20 minutes of exercise promotes beneficial effects on glycemic control, while in most previous studies the effect was observed with exercise sessions of 45-60 minutes [20, 23, 24, 30]. The reduction in glycemia occurred at the end of the exercise session and lasted up to 45 minutes, which did not occur in the control session (Table 3), suggesting an increase in blood glucose uptake by skeletal muscle as a result of the exercise [40].

Although we did not propose to investigate the intracellular processes of glucose uptake, we speculate that physical exercise, by promoting increases in blood circulation and hyperemia in muscle tissue, coincided with increased glucose delivery to active muscles. This response favors glucose uptake by myocytes, possibly through the release of Ca^2+^ from the sarcoplasmic reticulum, which stimulates mitogen activated phosphate kinases and the release of nitric oxide, which increases PI3K and AKT1 phosphorylation and results in glucose transporter 4 translocation [41, 43]. A previous study by our group [43] showed that individuals with T2D exhibit increased nitric oxide levels after 20 minutes of moderate exercise. This may have been a factor contributing to the observed increase in glucose uptake. In addition, our participants showed a reduction in the insulin/glucose ratio at 15 minutes of recovery after only the exercise session, reflecting an improved insulin sensitivity, as observed in other studies [22, 44].

After dextrose consumption, glycemia significantly increased in both experimental sessions, with no significant inter-regimen differences in terms of glycemia and insulinemia. Comparable results were obtained with respect to insulin/glucose ratio. Larsen et al. [23, 24] showed similar results regarding the AUCs of total glycemia and insulinemia as well as kinetics for individuals with T2D after consuming a meal containing all macronutrients 2.5 hours after a moderate aerobic exercise session (53% VO_2peak_) of 45 minutes (versus 20 minutes in this study). In another study [24], individuals with T2D showed no changes in glycemic and insulinemic responses to the first meal subsequent to a 46-minute session of interval exercise (4 series of 3 minutes at 57% VO_2peak_ and 4 minutes at 98% VO_2peak_ with 6 minutes of rest). The results of other studies are consistent with our results, since a longer exercise session (45 minutes at 60% VO_2peak_) had no effect on glycemic and insulinemic responses to subsequent meals eaten 3 or 4 hours after exercise in individuals with metabolic syndrome [21]. Moreover, a continuous exercise session of 30 minutes (75% VO_2peak_) or 20 min of interval exercise (125% VO_2peak_) did not attenuate glycemic and insulinemic responses in the OGTT performed 12 and 16 hours after the end of exercise by obese individuals [25].

In the study by Oberlin et al. [30], individuals with T2D underwent a control session and a treadmill exercise session with a duration of 60 min and an intensity between 60-75% of the heart rate reserve. Glycemia was monitored during the subsequent period, which included six standardized meals throughout the day for two days. The glycemia AUC was lower after subsequent meals, starting from the second one, when compared to the control day. These effects were not sustained on the second day of monitoring, suggesting that exercise must be performed on a daily basis to maintain these effects. In our study, we observed neither attenuated glycemic and insulinemic responses nor a smaller glycemia or insulinemia AUC after the consumption of a dextrose solution at 45 minutes of recovery after 20 minutes of exercise at moderate intensity (60-70% VO_2peak_).

Some methodological differences should be considered between our study and the abovereferenced studies. First, we used a cyclergometer, which only recruits muscle groups of the lower limbs. In addition, we adopted an exercise duration of only 20 min at a moderate intensity (90% LT), which is considered appropriate for individuals with low cardiorespiratory fitness such as our participants. The participants achieved reduced glycemia and a lower insulin/glucose ratio during 45 minutes of recovery. This suggests that acute physical exercise triggers the reduction in glycemia when performed in the postprandial condition (after breakfast) independently of the involved muscle mass, duration, or intensity, but does not attenuate the glycemic and insulinemic responses that follow the ingestion of carbohydrates 45 minutes after the end of moderate aerobic exercise. Similar results were found in individuals with metabolic syndrome [21] and obesity [25], but not in individuals with T2D [30], who exhibited a reduction in the total glycemia AUC after the second meal, ingested 60 min after moderate exercise.

Second, we chose to use a dextrose solution with a high glycemic index to induce more severe glycemic and insulinemic responses compared to foods of a low glycemic index [45]. The dextrose solution amount was adjusted according to the body weight (0.5 g/kg) to standardize the metabolic responses. This technique allows the assessment of glucose tolerance, the suggested confirmation of diabetes mellitus diagnosis, and the monitoring of disease progression [46].

Third, we speculate that the absence of attenuated glycemic and insulinemic responses in our study might be related to insufficient energy expenditure during the exercise session (~121.0 ± 7.8 kcal). The short exercise duration may not have sufficiently reduced the muscle glycogen reserve, as a result of which glucose uptake pathways were not sufficiently stimulated. We should expect to see significant reductions after exercise sessions with durations of > 45 minutes or intensities of > 75% VO_2peak_ [47]. However, such regimens would not be compatible with the low physical fitness level of our participants.

It is possible that a similar exercise duration (20 min) at a higher intensity would result in a more significant attenuation of glycemia increases following dextrose consumption, considering that a study by our group [16] performed with T2D showed a tendency to greater glycemic reductions following 20 min of more intense exercise (10% above LT) compared to a less intense exercise (90% LT). In another study [31], performed with pre-diabetic individuals, an intense (83% VO_2peak_) and short-duration exercise (23.8 ± 5 minutes) promoted larger reductions in postprandial glycemia and an increase of 85% in insulin sensitivity when compared to longer, moderate intensity exercise (40.1 ± 9 minutes at 50% VO_2peak_), which increased insulin sensitivity by 51%.

Additionally, we demonstrated the effectiveness of exercise in controlling glycemia. At the end of our recovery period (R135), eight participants performed 10 min of cyclergometer exercise at 90% LT, and the glycemia decreased from 230.46 ± 21.63 mg/dL to 185.92 ± 21.45 mg/dL, corresponding to a reduction of 44.5 ± 4.9 mg/dL.

In an elegant study by Holmstrup et al. [48], insulin-resistant individuals were engaged in a control session (no exercise), an exercise session (60 min at 60-65% of VO_2peak_) performed during the first waking hour in the morning right after the first meal of the day, and multiple walking exercise bouts lasting 5 minutes, performed at the beginning of each hour for 12 hours, while administering nutritionally-balanced liquid meals to monitor glycemia and insulinemia over that period. The authors observed that performing a multiple series of short physical exercise was more effective in reducing insulinemia and glycemia and insulinemia and glycemia AUCs in response to liquid meals. In that respect, our study confirmed that only 10-min exercise translated to glycemia reductions when performed 90 min after the consumption of a dextrose solution.

It is also important to underscore that no inter-regimen diferences were observed in the glycemic or insulinemic responses that followed the ingestion of a dextrose solution 45 min after exercise. Further investigation is needed to understand whether the ingestion of dextrose at different post-exercise moments, such as 10 or 15 min of post-excercise recovery, may induce any positive effect in attenuating glycemia or insulinemia following a carbohydrate challenge.

Finally, our study has some limitations. The absence of exercise and control sessions without carbohydrate consumption is one of the limiting factors that should be taken into account. In addition, insulin concentrations were not monitored at the same intervals as glycemia, although this difference did not impair analysis or outcomes.

In conclusion, a single exercise session of 20 minutes at moderate intensity (90% LT) promoted a decrease in glycemia and reduced the insulin/glucose ratio in T2D individuals during 45 minutes of post-exercise recovery, but did not affect the glycemia kinetics, the total glycemia and insulin AUCs, or the insulin/glucose ratio after consumption of a dextrose solution. Moderate exercise, even when carried out for only 10-20 min, was effective in reducing postprandial glycemia in T2D individuals and hence constitutes an important and accessible strategy for glycemic control in T2D individuals.
